# Russlands Wende nach Osten. Kleine Schritte statt umfassende Neuorientierung

**DOI:** 10.1007/s12399-020-00813-w

**Published:** 2020-10-06

**Authors:** Sebastian Hoppe, Vera Rogova

**Affiliations:** 1 Osteuropa-Institut (OEI), Freie Universität Berlin, Garystraße 55, 14195 Berlin, Deutschland; 2 Hessische Stiftung Friedens- und Konfliktforschung (HSFK), Leibniz-Institut, Baseler Straße 27-31, 60329 Frankfurt am Main, Deutschland

**Keywords:** Russland, Asien, Wirtschaftspolitik, Außenpolitik, Grand Strategy, Russia, Asia, Economic Policy, Foreign Policy, Grand Strategy

## Abstract

In den vergangenen Jahren hat Russland sein Engagement in Asien verstärkt, die Wende nach Osten wird gar als strategische Neuausrichtung des Landes diskutiert. Abseits der Rhetorik zeigt sich jedoch eine durchwachsene Bilanz. Nicht nur die Wirtschaftsaußenpolitik läuft mit ihrem Fokus auf Energie- und Rüstungsprojekte Gefahr, jene Strukturen zu reproduzieren, die Russlands außenpolitische Handlungsspielräume und ökonomische Entwicklung in der Vergangenheit eher behindert denn befördert haben. Auch der einstweilen erfolgreiche Ausbau des sicherheitspolitischen Engagements in Asien steht vor der Herausforderung, die sich abzeichnende Abhängigkeit gegenüber China zu gestalten.

## Abstract (en)

In recent years Russia has intensified its engagement in Asia. The Turn to the East is even being discussed as a strategic reorientation of the country. Apart from the rhetoric, however, the results are mixed. Russia’s foreign economic policy, with its focus on energy and armaments projects, runs the risk of reproducing the very structures that have limited the country’s foreign policy options and hindered economic development in the past. Furthermore, the expansion of Russia’s security-related engagement in Asia, while being currently successful, faces serious challenges in light of an emerging dependence on China.

## Einleitung

Russlands Selbstverständnis und Rolle im internationalen System befinden sich seit einiger Zeit im Wandel. Innenpolitisch wird dies von einer kontroversen Debatte über die großen Fragen nationaler Identität und globaler Ordnungsvorstellungen begleitet. Eine zentrale These ist dabei, dass die traditionelle Orientierung nach Europa und nach Westen an Bedeutung verliere, während die Zusammenarbeit mit Staaten in Zentralasien und der asiatischen Pazifikregion immer wichtiger werde.

Die Idee, Russland solle sich politisch wie ökonomisch stärker nach Osten orientieren, ist nicht neu. Bereits in den 1990er Jahren sprach sich der damalige Außenminister Jewgeni Primakow dafür aus, vor allem den Sicherheitsdialog mit China und Indien zu intensivieren und ein sogenanntes strategisches Dreieck Moskau-Peking-Neu-Delhi aufzubauen (Kommersant 1996). In den Folgejahren betonten auch der russische Präsident Wladimir Putin und Mitglieder seiner Regierung immer wieder, wie wichtig für Russland gute Beziehungen zu den aufstrebenden asiatischen Mächten, allen voran China, seien (Putin 2012a; Lawrow 2006). Das Projekt erhielt mit Russlands politisch-militärischem Eingreifen nach dem Kiewer Euromaidan im Jahr 2014 und den sich anschließend massiv verschlechternden Beziehungen zu den westlichen Staaten für Moskau eine neue Relevanz und Dringlichkeit. Es zeigt sich allerdings, dass das innenpolitische Ziel, die ökonomische Entwicklungsdynamik Asiens für eine nachhaltige regionale Entwicklung zu nutzen, bisher nicht erreicht werden konnte. Außenpolitisch sind im Verhältnis zu China durchaus Fortschritte zu verzeichnen, auch wenn vorerst offenbleiben muss, als wie nachhaltig sich diese tentative Annäherung angesichts konkurrierender Integrationsprojekte für die Region erweisen wird.

## Die russische Debatte und das strategische Konzept

Unter dem Oberbegriff des poworot na Wostok^1^ wird in der russischen politischen und akademischen Debatte eine Vielzahl unterschiedlicher Konzepte, Entwicklungen und Zielsetzungen diskutiert.^2^ Zum einen könne dieser, so der Ehrenvorsitzende des russischen Rates für Außen- und Verteidigungspolitik Sergei Karaganow, als zwingende Folge machtpolitischer Verschiebungen in der asiatischen Region verstanden werden: „Die heutige und zukünftige Realität diktieren die Notwendigkeit einer integrierten Entwicklungsstrategie für Sibirien, das zentrale Eurasien und den Fernen Osten gemeinsam mit den asiatischen Partnern. Möglicherweise kann das auch neue Horizonte für Europa eröffnen, das seine Dynamik verloren hat“ (Karaganow 2015, eigene Übersetzung; Kaschin 2016). Die rasante wirtschaftliche Dynamik Chinas, die in einigen Branchen beachtlichen sozioökonomischen Entwicklungserfolge Indiens und Vietnams sowie die Nähe zu den hochentwickelten Ökonomien Japans und Südkoreas haben dazu beigetragen, dass sich für Russland breite Möglichkeiten für neue Handelsbeziehungen und Investitionen eröffnet haben.

Mit dem ökonomischen geht wiederum ein politischer Bedeutungsgewinn der Region einher. Hier ist vor allem das zunehmende Engagement Chinas zu verzeichnen, auch im Rahmen der einschlägigen Regionalorganisationen wie der Shanghai Cooperation Organisation (SCO), der Association of Southeast Asian Nations (ASEAN) oder zuletzt mit der One Belt One Road Initiative (OBOR). Dieses globale Investitionsprojekt unter chinesischer Führung und mit einem geplanten Volumen von über einer Billion US-$ könnte schließlich über 60 Länder in Zentralasien, Südostasien, am Persischen Golf und bis hin nach Afrika und Europa umfassen (Huang 2016; Zhou und Esteban 2018). Für Russland, das sich gerade in Zentralasien lange Zeit als Hegemon betrachtete, bedeutet der rasche Aufstieg Chinas eine strategische Herausforderung, auf die die politische Führung und die Wirtschaft Antworten finden müssen (Cooley 2019).

Eine zweite Dimension des poworot besteht in der bewussten strategischen Entscheidung der russischen Führung, den wirtschaftlichen Aufstieg Asiens für entwicklungspolitische Ziele zu nutzen, vor allem im Hinblick auf die innerrussische regionale Entwicklung (Blakkisrud 2018). Zur Zeit der Präsidentschaft Dmitri Medwedews, der seine Amtszeit ins Zeichen der Modernisierung und sozioökonomischen Entwicklung Russlands gestellt hatte, besaß die Hinwendung nach Osten somit eine starke innenpolitische Dimension. Die engere wirtschaftliche Zusammenarbeit mit asiatischen Staaten wurde als eine Möglichkeit aufgefasst, den ökonomisch rückständigen Regionen Russlands jenseits des Urals – also Sibirien und dem russischen Fernen Osten – zum Aufschwung zu verhelfen. So betonte Medwedew, dass „die Integration mit den Ländern der asiatisch-pazifischen Region ein riesiges Potenzial für die Wirtschaftsentwicklung des Fernen Ostens und ganz Russlands bietet“ (Medwedew 2010, eigene Übersetzung).

Ebenso steht der poworot im Kontext globaler Ordnungsvorstellungen der außenpolitischen Eliten Russlands. Schon länger wird hier das Ende der unipolaren, vom Westen und vorrangig von den USA dominierten internationalen Ordnung diagnostiziert. Eine neue, multipolare Weltordnung sei im Entstehen, so Putin bereits in einem viel beachteten Artikel aus dem Jahr 2012 (Putin 2012b). In dieser spiele auch Russland eine neue Rolle, je nach Auslegung als eigenständiger, regionaler Machtpol oder aber als Verbindungsglied zwischen Europa und den von China dominierten, asiatischen Regionen (Makarychev und Morozov 2011; Chebankova 2017). Eingang fand diese Perspektive beispielsweise in die 2013 veröffentliche Konzeption der Außenpolitik Russlands (MFA 2013).

Durch die dramatisch verschlechterten wirtschaftlichen und diplomatischen Beziehungen zur EU und zu den USA im Nachgang der Ukraine-Krise im Jahr 2014 erhielt diese Debatte jedoch eine neue Dringlichkeit. Der russischen Regierung war nun daran gelegen zu signalisieren, dass das Land durchaus Alternativen bei der Wahl seiner internationalen Partner habe und sich daher von westlicher Kritik oder den Sanktionen nicht werde beeindrucken lassen. Ein unmittelbares Ergebnis dieser Intensivierung war etwa der schnelle Verhandlungsabschluss zwischen China und Russland noch im Mai 2014 über ein langfristiges Lieferabkommen für Erdgas. Putin persönlich reiste nach Shanghai, um den sich zuvor über zehn Jahre hinziehenden Prozess zu einem Abschluss zu bringen (Hornby et al. 2014).

Diese Entwicklungen gingen schließlich auch mit ideellen Verschiebungen einher (Lewis 2018). Das lange Zeit vorherrschende Selbstverständnis der staatlichen Elite, aber auch der breiten Bevölkerung, Russland sei ein politisch, wirtschaftlich und gesellschaftlich europäisches Land, in Verbindung mit einer Skepsis gegenüber asiatischen Ländern, begann sich zu wandeln. Während beispielsweise im März 2006 bei einer Umfrage der Stiftung Öffentliche Meinung (FOM) 13 % angaben, „schlecht“ über China zu denken, 51 % „gleichgültig“ und 32 % „positiv“, waren es im April 2014 respektive 3 %, 46 % und 42 % (FOM 2014, eigene Übersetzung). Das Levada-Zentrum ermittelte im September 2018 sogar 75 %, die „sehr gut“ oder „gut“ zu China standen, während nur 13 % „schlecht“ oder „sehr schlecht“ eingestellt waren (Levada o.J., eigene Übersetzung). Zwar sanken seitdem diese Zustimmungswerte, nicht zuletzt befeuert durch Skepsis gegenüber China im Zuge der Covid-19-Pandemie, die jedoch vorerst keinen Einfluss auf die Dynamik der bilateralen Beziehungen zu nehmen scheint (Zuenko 2020).

## Die wirtschaftliche Dimension

Einen zentralen Bestandteil des poworot stellen die wirtschaftlichen Beziehungen zu Ländern im asiatischen Pazifikraum dar, deren Intensivierung vielfach als Ziel verkündet worden war (Putin 2013). Entsprechend lässt sich sowohl vor als auch nach 2014 eine beachtliche staatliche Aktivität beobachten, die sich auf Sibirien und den Fernen Osten sowie international auf andere Länder der Region richtet. Die Entwicklung der Regionen wird dabei als Grundlage für erfolgreiche Außenwirtschaftsbeziehungen betrachtet, sodass sich hier innen- und außenpolitische Überlegungen verbinden (Larin 2017).

Zumindest in der Theorie beruht die Strategie der russischen Regierung dabei auf dem Entwicklungsmodell einer offenen, auf ausländische Direktinvestitionen und Exporte ausgerichteten Wirtschaft, die durch staatliche Maßnahmen zur Verbesserung der Investitionsbedingungen flankiert werden soll. Bereits im Mai 2012 wurde hierzu ein Ministerium für die Entwicklung des Fernen Ostens gegründet, das bis 2013 eine überarbeitete Entwicklungsstrategie für die Region vorlegte und die initiierten Programme verwalten sollte. Das Dokument wurde in den folgenden Jahren immer wieder überarbeitet und setzte zunehmend auf Diversifizierung und die Förderung innovativer Technologien in der Region Ferner Osten, während natürliche Ressourcen als die für Russland dominierenden Exportgüter in den Hintergrund treten sollten (Government of the Russian Federation 2013; Fortescue 2016).

Begleitet wurden diese Absichtserklärungen von unterschiedlichen Maßnahmen, die unter anderem mithilfe staatlicher Subventionen Investitionen aus dem In- und vornehmlich asiatischen Ausland in die Region locken sollten. So wurden im Dezember 2014 sogenannte Territorien der überholenden Entwicklung (TOR) eingerichtet, die Erleichterungen wie vergünstigte Kredite, reduzierte Mieten, Steuererleichterungen oder vereinfachte administrative Prozesse für mögliche Investoren vorsehen (Federal Law 2014). Der Schwerpunkt der Förderung liegt dabei auf Unternehmen aus technologisch-innovativen Sektoren. Bis heute wurden 18 TORs eingerichtet, darunter 2015 der Freie Hafen Wladiwostok; die Investoren kommen vor allem aus Russland, aber auch aus China, Japan, Südkorea oder Australien.^3^ Auch im Bereich Humankapital wurde die Regierung aktiv. Nachdem die Region lange Zeit eine negative Bevölkerungsentwicklung verzeichnet hatte, wurde 2016 das Programm Das fernöstliche Hektar ins Leben gerufen: Alle russischen Staatsangehörigen können kostenlos ein Hektar Land in der Region Ferner Osten erhalten, wenn sie sich dort langfristig niederlassen (Federal Law 2016).

Zentral war gleichfalls der Ausbau der Infrastruktur, die große Schwachstelle des Fernen Ostens. Nicht zuletzt aufgrund enormer Entfernungen und schwieriger Wetterbedingungen ist das Interesse privater Investoren gering, so dass die russische Regierung verschiedene, staatlich geförderte Programme ankündigte (Government of the Russian Federation 2016). Mit diesen Mitteln sollte z. B. eine Verbesserung von Hafeninfrastruktur und Straßen erreicht sowie das Schienennetz in der Region modernisiert werden. Schließlich wirbt Russland aktiv um Investoren aus dem In- und Ausland. Den symbolträchtigen Auftakt dazu bildete der Gipfel der Asiatisch-Pazifischen Wirtschaftsgemeinschaft (Asia-Pacific Economic Cooperation, APEC) in Wladiwostok im September 2012. Auch in den folgenden Jahren zeigte sich die Stadt als Zentrum insbesondere wirtschaftspolitischer Diplomatie und ist u. a. jährlicher Austragungsort des 2015 ins Leben gerufenen Eastern Economic Forum oder des International Economic Business Congress, der seit 2012 Spitzenpersonal großer Unternehmen aus dem Föderationskreis Ferner Osten und der asiatischen Pazifikregion versammelt (Troyakova 2018, S. 43–44).

Es zeigt sich demnach, dass auf der Planungsebene durchaus ein Umdenken stattfand, das als Hinwendung nach Osten interpretiert werden kann. Gemessen an den vielfältigen Strategiepapieren und Aktivitäten konnte die russische Regierung ihre selbstgesteckten Ziele im entwicklungspolitischen Bereich jedoch nur bedingt erreichen (Spivak 2019). Die Implementierung und langfristige Wirkung der politischen Maßnahmen, die auf regionale Entwicklung ausgerichtet waren, werden durch die Besonderheiten des russischen Wirtschaftssystems erheblich eingeschränkt – ähnlich wie es in der Vergangenheit bereits Medwedews Modernisierungsagenda ergangen war (Gel’man 2017). Entgegen den Ankündigungen der russischen Regierung, gerade privatwirtschaftliche Initiative sowie den Export von Waren außerhalb des Rohstoffsektors stärken zu wollen, scheinen vom poworot erneut jene staatsnahen Sektoren zu profitieren, die bereits eine dominante Stellung in der politischen Ökonomie Russlands einnehmen: natürliche Ressourcen oder die Rüstungsindustrie.

Diese Ausrichtung ist auch durch die russischen Außenwirtschaftsbeziehungen in der Region bestimmt. Vorrangig ist hier China zu nennen, Russlands größter Handelspartner.^4^ Die im weltweiten Vergleich immer noch schnell wachsende Ökonomie ist auf Erdgasimporte angewiesen und stellt einen zunehmend wichtigen Absatzmarkt für Russlands Energieexporte dar (Clemente 2016). So entfielen noch im Jahr 2008 52 % der chinesischen Importe aus Russland auf diesen Bereich, 2018 waren es bereits 73 % (World Bank o.J.). Im Frühjahr 2014 hatten Gazprom und die China National Petroleum Corporation (CNPC) einen Vertrag unterzeichnet, der über einen Zeitraum von 30 Jahren Gaslieferungen von jährlich 38 Mrd. m^3^ vorsieht. Hierfür begann Gazprom im September 2014 mit dem Bau einer neuen Pipeline mit einer Länge von 2200 km, die Erdgas vom russischen Feld in Tschadinskoje bis nach Blagoweschtschensk an der Grenze zu China transportieren soll. Die erste Lieferung über die Sila Sibiri-Pipeline^5^ fand planmäßig am 2. Dezember 2019 statt. Xi und Putin tauschten zu diesem Anlass freundliche Worte in einer Videokonferenz aus (Schweizer 2019).

Weitere Projekte sind in Planung. Gazprom und CNPC verhandeln aktuell über das riesige Pipeline-Projekt Sila Sibiri 2, das zukünftig Fördergebiete im Norden Sibiriens mit Westchina verbinden könnte. Im Mai 2020 hatte der Vorstandsvorsitzende Alexei Miller den Beginn der Planungsphase des Projekts verkündet (Eurasia Daily 2020). Im Bereich Flüssiggas (LNG) sind chinesische Investoren zum Beispiel über den CNPC (20 % Anteile) sowie den Silk Road Fund (9,95 %) am Projekt Yamal LNG beteiligt, das die riesigen Gasvorkommen in der Arktis erschließen will und dabei vor allem den lukrativen asiatischen Markt im Blick hat (Ballin 2018).^6^

Einerseits zeigen diese Großprojekte, dass die Kooperation auf Dauer gestellt werden soll. Damit könnte der asiatische Energiemarkt langfristig die zunehmend angespannten Wirtschaftsbeziehungen zu europäischen Staaten (siehe die Kontroverse um Nord Stream 2) wenn nicht ersetzen, so doch zumindest ergänzen. Auf der anderen Seite birgt das (ökonomische) Machtgefälle zwischen Russland und China, das sich in Zukunft wohl noch weiter zu Ungunsten Russlands verschieben wird, strategische Risiken und Konkurrenzverhältnisse. Während etwa Russlands eigenes Integrationsprojekt der Eurasischen Wirtschaftsunion in den rund fünf Jahren seines Bestehens kaum Erfolge zeitigen konnte, hat China seinen Einfluss in der Region kontinuierlich ausgebaut (Stronski und Ng 2018).

Die russische Führung scheint sich dessen bewusst zu sein: So müssen die Bemühungen um eine engere ökonomische Kooperation mit China auch als der Versuch der Einhegung interpretiert werden, um Einflussmöglichkeiten in der asiatischen und auch zentralasiatischen Region nicht gänzlich einzubüßen (Skalamera 2017).

## Ausbau des sicherheitspolitischen Engagements in Asien

Zum Gesamtbild des poworot gehört auch das verstärkte Interesse Russlands, die sicherheitspolitische Kooperation mit den entscheidenden Ordnungsmächten in Asien, allen voran China, Japan und Indien, zu intensivieren. Einerseits erhofft sich der Kreml hierdurch eine Verbesserung auch der Außenwirtschaftsbeziehungen, wie etwa nach der Finalisierung der russisch-chinesischen Grenze im Jahr 2004 (Ambrosio 2017, S. 134). Andererseits steht Russland vor der Herausforderung, sensibel mit den teils konfliktiven Sicherheitsinteressen einer ganzen Reihe staatlicher Akteure in der Region sowie dem Ordnungsanspruch der USA umgehen zu müssen.

Als Beispiel kann der seit mehr als 70 Jahren schwelende Territorialstreit um die Inselgruppe der Kurilen zwischen Russland und Japan angeführt werden. Obwohl die diplomatischen Aktivitäten der vergangenen Jahre nahelegen, dass der Wille zur Beilegung des Konflikts auf beiden Seiten vorhanden ist (TASS 2019), steht eine solche Lösung immer noch vor enormen Hindernissen. Den Aussagen Putins im Herbst 2018, man könne den Territorialkonflikt durch einen gemeinsamen Friedensvertrag beilegen, müssen die Prognosen des militärisch-industriellen Komplexes in Russland gegenübergestellt werden, die für den Fall einer vollständigen oder teilweisen Abtretung der Inseln die Stationierung amerikanischer Militärinfrastruktur in unmittelbarer Nähe der russischen Pazifikflotte prophezeien (Panda 2019). Hinzu kommt, dass Putin eine solche Gebietsabtretung an die Bereitschaft der russischen Öffentlichkeit gebunden hat (Soldatkin 2019), die sich in einer Umfrage vom November 2018 jedoch mit 72 % gegen eine Abtretung auch nur eines Teils der Inseln an Japan aussprach (Levada 2018). Gleichzeitig entwickelt sich mit der enger werdenden russisch-chinesischen Militärkooperation ein mögliches Hindernis für einen Ausgleich mit Japan. So hielt die russische Pazifikflotte im August 2015 zum ersten Mal ein Manöver mit chinesischen Einheiten im Japanischen Meer ab. Zuvor war es stets das Ziel Moskaus gewesen, militärische Manöver zu vermeiden, die in Tokio als Provokation aufgefasst werden könnten (Reuters 2015).

Dass Russland im großen Stil Waffensysteme verschiedenster Art an asiatische Länder liefert, ist kein Novum. So gingen 56 % der russischen Waffenexporte zwischen 2000 und 2016 allein an Indien und China; Asien insgesamt war für 70 % der Exporte verantwortlich. In der Tat herrscht auf diesem Gebiet Interdependenz: Fast die Hälfte (43,1 %) aller Waffenimporte in den asiatischen Pazifikraum kommen aus Russland (Connolly und Sendstad 2017, S. 11). Während jedoch in der Vergangenheit insbesondere der Generalstab der russischen Armee sowie die Geheimdienste gegen den Export erweiterter Waffentechnologien vor allem an China waren, kam es in den letzten Jahren zu einer Aufweichung dieser Bestimmungen. Glaubt man dem russischen Militär nahestehenden Expert*innen, so gab es ein generelles, informelles Verbot des Exports der entwickeltsten Waffensysteme von 2004 bis 2014 (Financial Times 2018). Unter anderem die Versicherungen der chinesischen Seite, man werde sich zukünftig an das bereits 2008 geschlossene Abkommen über geistige Eigentumsrechte im Rahmen von Waffenexporten halten, führten jedoch letztendlich zur Aufhebung dieser selbst auferlegten Beschränkung (Røseth 2018, S. 8). Die von Putin im Jahr 2015 genehmigte Lieferung der modernen S‑400-Langstrecken-Boden-Luft-Raktensystems an die chinesische Volksbefreiungsarmee zeigt (Gady 2018), dass sich auf diesem Feld vorerst Export- gegen Sicherheitsinteressen durchgesetzt haben. Nichtdestotrotz bestehen nach wie vor Spannungen zwischen den Interessen der russischen Rüstungsindustrie und dem sicherheitspolitischen Ziel, regionale Militärgleichgewichte aufrechtzuerhalten, die jedoch perspektivisch Gefahr laufen, von intensivierten Rüstungsexporten verändert zu werden. So kann das S‑400-System nicht nur von chinesischem Territorium aus Ziele im russischen Luftraum erreichen, auch die vom russischen Staat bereits zur Lieferung freigegebenen SU-35 Kampfjets sowie U‑Boote der Amur-Klasse wären im Konfliktfall gegen die eigene Pazifikflotte einsetzbar. Es zeichnet sich dennoch ab, dass sich in Teilen der russischen Elite das wahrgenommene Gefahrenpotential Chinas relativiert hat, was einerseits mit den chinesischen Zugeständnissen, andererseits jedoch auch mit der Notwendigkeit zusammenhängen mag, sich vor dem Hintergrund sinkender Exporteinnahmen mit westlichen Staaten neue Exportmärkte zu erschließen (Røseth 2018, S. 8–9).

Im militärischen Bereich arbeitet Moskau kontinuierlich daran, die eigene Präsenz im asiatischen Pazifikraum auszubauen. Im September 2018 hielt das russische Militär die Übung Wostok 2018^7^ ab, das größte Militärmanöver seit 1981 mit nominell 291.000 beteiligten russischen Soldaten. China war an diesem Manöver mit 3200 Soldaten, 1000 Fahrzeugen sowie 30 Flugzeugen beteiligt, nahm jedoch lediglich an gemeinsamen Übungen in der Region Transbaikalien teil. Bereits im Rahmen der SCO hatten Russland und China gemeinsame Übungen abgehalten, Wostok hingegen simulierte zum ersten Mal gemeinsame Kriegshandlungen und muss demnach als qualitative Intensivierung der militärischen Kooperation betrachtet werden (Yang 2018). Hinzu kommt ein im April 2015 geschlossenes Rahmenabkommen über Angelegenheiten der internationalen Informationssicherheit, das die beiderseitige Verpflichtung enthält, gegeneinander keine Cyberattacken durchzuführen und zusammen gegen destabilisierende Technologien vorzugehen, die bspw. im Rahmen von sogenannten Farbrevolutionen eingesetzt werden könnten (Klein und Westphal 2015, S. 2–3).

Zwar ist die Militärpräsenz Russlands in Asien keineswegs mit jener der USA vergleichbar. Allerdings gelingt es dem russischen Militär durch selektive Maßnahmen – gemeinsame Manöver mit China, Indien und Japan, die Nutzung indischer Militärhäfen, Gespräche mit dem vietnamesischen Militär und nicht zuletzt umfangreiche Rüstungsexporte –, die eigene Position sukzessive auszubauen (Cobb 2017). Jenseits der militärischen und politischen Zusammenarbeit mit China kann im Zuge des poworot beobachtet werden, wie entweder, wie im Fall der russisch-vietnamesischen Beziehungen, aus Sowjetzeiten stammende, militärisch-diplomatische Gesprächskanäle intensiviert, oder jene Handlungsspielräume bedient werden, die sich aus den Bestrebungen einiger asiatischer Länder ergeben, sich nicht einseitig in die chinesische Hegemonie begeben zu wollen. Dieses Vorgehen und nicht zuletzt die Rüstungsexporte an miteinander im Konflikt liegende Staaten bedeuten dabei einen ständigen diplomatischen Balanceakt, den Moskau bis jetzt jedoch ohne größere Flurschäden zu bewältigen scheint.

## Fazit. Eine strategische Neuausrichtung nach Osten?

Insgesamt ergibt sich eine durchwachsene Zwischenbilanz der russischen Wende nach Osten. Einerseits zeichnen sich einige erfolgreiche Projekte ab, etwa in der Energie- und der militärischen Kooperation, die zeigen, dass der poworot mehr als bloße Rhetorik ist. Gerade in der Sicherheitspolitik lässt sich eine vor wenigen Jahren noch nicht gekannte Bereitschaft verzeichnen, gegenseitiges Vertrauen aufzubauen und festgefahrene Konflikte aufzulösen. Auf politischer Ebene existiert in Russland zweifellos der Wille, eine Strategie im Umgang mit den Partnern im asiatisch-pazifischen Raum zu finden.

Der ökonomische Druck der anhaltenden Wirtschaftskrise und die Auswirkungen westlicher Sanktionen mögen zu dieser erhöhten politischen Kompromissbereitschaft der russischen Führung beigetragen haben. Somit werden auch die Entwicklung der Beziehungen zur EU und den USA sowie die Zukunft der Sanktionen einen Einfluss darauf haben, als wie stark und nachhaltig sich der Trend Richtung Asien erweist. Die Paradoxie der gegenwärtigen Situation besteht darin, dass sie die politischen Beziehungen zu einigen asiatischen Partnern zu verbessern scheint, während eine Vertiefung der ökonomischen Integration im asiatisch-pazifischen Raum einfacher ohne Sanktionsregime zu realisieren wäre (Vakulchuk 2018).

Die Bilanz des selbstgesetzten Ziels, die neue außenpolitische Dynamik vor allem auf die Diversifizierung und Modernisierung der Wirtschaft in den fernöstlichen Regionen Russlands zu gründen, fällt wenig erfolgreich aus. Die mit neuen Großprojekten fortgeschriebene Ausrichtung auf Energieexporte ist mit Risiken verbunden, da sie mit einer ökonomischen (und letztlich auch politischen) Abhängigkeit von globalen Rohstoffpreisen und großen Abnehmerländern einhergeht. Gerade in Bezug auf China ist zu befürchten, dass sich das für Russland ungünstige Kräfteverhältnis noch weiter verschlechtern wird. Letztlich wird es vom außenpolitischen Management der russisch-chinesischen Asymmetrie und der Umsetzung jener Vorhaben abhängen, die die russische Wirtschaftsstruktur diversifizieren und kompatibel mit asiatischen Wertschöpfungsketten machen sollen, welchen Platz Russland in der zukünftigen Ordnung der asiatisch-pazifischen Großregion einnehmen wird.

## Footnotes

1 Zu Deutsch: Wende nach Osten.

2 Im laufenden Text wurde zur Umschrift aus dem Russischen die allgemein gebräuchliche Duden-Transkription verwandt. In den Literaturhinweisen und im Literaturverzeichnis wurde die bibliothekarische Transliteration genutzt.

3 Eine vollständige Liste findet sich unter: https://minvr.ru/activity/territorii-operezhayushchego-razvitiya/ (Zugegriffen: 9. Juli 2020).

4 Während im Bereich der Importe China schon länger den ersten Platz belegte, wurde das Land 2017 auch zum größten Abnehmer russischer Exporte. Siehe hierzu: Russian Federation Trade Summary 2017. https://wits.worldbank.org/CountryProfile/en/Country/RUS/Year/2017/SummaryText (Zugegriffen: 9. Juli 2020).

5 Zu Deutsch: Kraft Sibiriens-Pipeline.

6 Homepage des Projekts: http://yamallng.ru/project/about/ (Zugegriffen: 9. Juli 2020).

7 Zu Deutsch: Osten 2018.
